# Ligament mechanics of ageing and osteoarthritic human knees

**DOI:** 10.3389/fbioe.2022.954837

**Published:** 2022-08-23

**Authors:** Abby E. Peters, Brendan Geraghty, Karl T. Bates, Riaz Akhtar, Rosti Readioff, Eithne Comerford

**Affiliations:** ^1^ Department of Mechanical, Materials and Aerospace Engineering, School of Engineering, University of Liverpool, Liverpool, United Kingdom; ^2^ Institute of Life Course and Medical Sciences, University of Liverpool, Liverpool, United Kingdom; ^3^ Medical Research Council Versus Arthritis Centre for Integrated Research Into Musculoskeletal Ageing (CIMA), University of Liverpool, Liverpool, United Kingdom; ^4^ Institute of Medical and Biological Engineering, School of Mechanical Engineering, Faculty of Engineering, University of Leeds, Leeds, United Kingdom; ^5^ School of Dentistry, University of Liverpool, Liverpool, United Kingdom; ^6^ School of Veterinary Science, University of Liverpool, Liverpool, United Kingdom

**Keywords:** human knee ligament, material characterisation, knee osteoarthitis, anterior cruciate ligament, posterior cruciate ligament, lateral collateral ligament, medial collateral ligament, ageing

## Abstract

Knee joint ligaments provide stability to the joint by preventing excessive movement. There has been no systematic effort to study the effect of OA and ageing on the mechanical properties of the four major human knee ligaments. This study aims to collate data on the material properties of the anterior (ACL) and posterior (PCL) cruciate ligaments, medial (MCL) and lateral (LCL) collateral ligaments. Bone-ligament-bone specimens from twelve cadaveric human knee joints were extracted for this study. The cadaveric knee joints were previously collected to study ageing and OA on bone and cartilage material properties; therefore, combining our previous bone and cartilage data with the new ligament data from this study will facilitate subject-specific whole-joint modelling studies. The bone-ligament-bone specimens were tested under tensile loading to failure, determining material parameters including yield and ultimate (failure) stress and strain, secant modulus, tangent modulus, and stiffness. There were significant negative correlations between age and ACL yield stress (*p = 0.03*), ACL failure stress (*p = 0.02*), PCL secant (*p = 0.02*) and tangent (*p = 0.02*) modulus, and LCL stiffness (*p = 0.046*). Significant negative correlations were also found between OA grades and ACL yield stress (*p = 0.02*) and strain (*p = 0.03*), and LCL failure stress (*p = 0.048*). However, changes in age or OA grade did not show a statistically significant correlation with the MCL tensile parameters. Due to the small sample size, the combined effect of age and the presence of OA could not be statistically derived. This research is the first to report tensile properties of the four major human knee ligaments from a diverse demographic. When combined with our previous findings on bone and cartilage for the same twelve knee cadavers, the current ligament study supports the conceptualisation of OA as a whole-joint disease that impairs the integrity of many peri-articular tissues within the knee. The subject-specific data pool consisting of the material properties of the four major knee ligaments, subchondral and trabecular bones and articular cartilage will advance knee joint finite element models.

## Introduction

Tensile properties of the anterior cruciate ligament (ACL), posterior cruciate ligament (PCL), medial collateral ligament (MCL) and lateral collateral ligament (LCL) have been explored by numerous researchers (Noyes & Grood, 1976; Woo et al., 1991; Race & Amis, 1994; Robinson, Bull & Amis, 2005; Bonner et al., 2015; Smeets et al., 2017; Cho & Kwak, 2020; Patel et al., 2021), providing vital information on their structural and mechanical properties. There are data for all four ligaments from varying specimens in previous studies; however, there is marked variability in the reported values, likely due to variations in testing techniques and donor demographics, making it challenging to understand the whole-joint function (Peters et al., 2018a). To date, very few studies have explored all four ligaments from the same donor (donors were limited to healthy knee joints), with data suggesting higher stiffness and failure load in the cruciate ligaments compared to the collateral ligaments (Trent, Walker & Wolf, 1976; van Dommelen et al., 2005).

The lack of consistent healthy baseline measurements means our understanding of how tensile properties of all four ligaments within the same knee joint change with ageing or disease is presently unclear (Peters et al., 2018a). Structural and functional capabilities are known to decline with age in the ACL, in particular, a decrease in ultimate failure load from older donors (67–90 years) when compared to donors between 40 and 50 years old and younger donors (22–35 years) (Woo et al., 1991). This decline in properties is also reflected at a cellular level in ligaments such that ACL histological parameters showed an increase in tissue degeneration with age (Hasegawa et al., 2012). However, any differences in material properties in the PCL, MCL and LCL are yet to be systematically correlated with different age categories. Changes to integrity and tensile properties not only leave ligaments vulnerable to further injury but also affect the peri-articular tissues leading to muscle weakening through immobility and whole-joint disruption, including the development of osteoarthritis (OA) (Manninen et al., 1996; Rousseau & Garnero, 2012; Simon et al., 2015). In addition, our knowledge about the effect of OA on the tensile properties of the knee joint ligaments is limited, with current studies focusing primarily on histological analyses. There is evidence showing impaired integrity of the ACL and PCL during total knee replacements in the presence of OA and with age (Mullaji et al., 2008; Hasegawa et al., 2012).

Previously, we systematically investigated the effect of age and OA on the mechanical properties of bone and cartilage in human knee joints for the first time in the same donor (Peters et al., 2018b). Here, we have employed the same human cadavers to study the ligaments, which will allow 1) the first assessment of changes in the mechanical behaviour of ligaments due to ageing and OA, 2) ligament data to be combined with bone and cartilage trends from the same specimen to give a fuller picture of the multi-tissue joint (whole-joint) changes with age and OA, and 3) the subject-level data to be used in the future development of subject-specific OA knee joint computer models. Thus, this study aimed to obtain data on tissue-level material characteristics of cadaveric human knee joint ligaments with a wide span of age and OA grades and correlate these to age and OA grade as univariate parameters. The following objectives were performed to fulfil the aim of this study:

1 To harvest the four major knee joint ligaments (ACL, PCL, LCL and MCL) as bone-ligament-bone specimens and measure undeformed geometrical parameters of the ligaments.
2 To apply physiologically relevant tensile loads on the ligaments and determine their mechanical responses.
3 To analyse the ligaments’ tensile characteristics and tests their correlations with age and OA.

## Materials and methods

### Specimens

Fresh-frozen human cadaveric knee joints were sourced from Science Care (Phoenix, Arizona, United States) via Newcastle Surgical Training Centre (Newcastle upon Tyne, NE7 7DN, United Kingdom), and consents were obtained and held by Science Care. The knee cadavers were from humans aged 31–88 years (*n* = 12; four female and eight male) (Supplementary Table S2) as reported in our previous study (Peters et al., 2018b). Ethical permission for using the human cadaveric materials was sponsored by the University of Liverpool and granted by the National Research Ethics Service (15/NS/0053), who approved all protocols. All experiments were performed following relevant guidelines and regulations.

**FIGURE 1:**
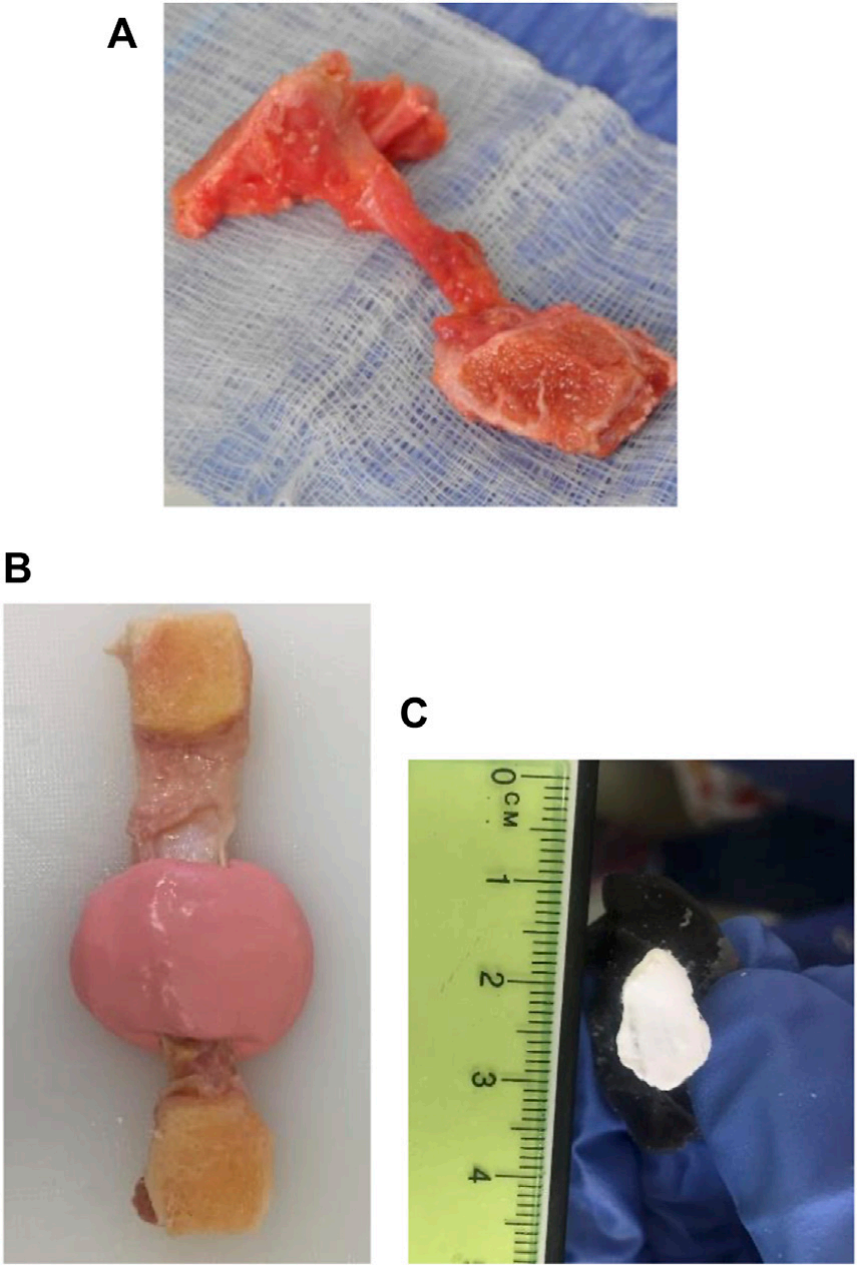
Bone-ligament-bone preparation and method for measuring the middle cross-sectional area of knee joint ligament specimens. **(A)** Bone-ligament-bone specimen. **(B)** Ligament encased in the impression material. **(C)** A polymethyl-methacrylate cast of a ligament photographed for cross-sectional area measurement.

Cadaver limbs were initially frozen at -20°C and thawed at 3–5°C for 5 days before dissection. During dissection, cadavers were photographed and graded for OA using the International Cartilage Repair Society (ICRS) (Supplementary Table S1) as reported in our previous study (Peters et al., 2018b). Four bone-ligament-bone specimens were harvested from each cadaver using a low-speed oscillating saw (deSoutter Medical, Bucks, UK) (Figure 1A). Extracted specimens were stored at -20°C before thawed for 24 h at 3–5°C and submerged in phosphate-buffered saline (PBS). Overall the specimens underwent two freeze-thaw cycles, which have previously been shown not to affect ligament and tendon material properties (Woo et al., 1986; Moon et al., 2006; Huang et al., 2011; Jung et al., 2011; Peters et al., 2017). Specimen numbers are consistent with those in Peters et al. (2018b), allowing the matching of ligament properties presented here with previously reported cartilage and bone data from the same individuals (Supplementary Materials (Ligament Raw Data. xlsx)).

### Length and cross-sectional area measurements

Prior to mechanical tests, lengths of the ligaments were collected, and these were determined as the distance between the bone attachment areas using Vernier callipers (D00352, Duratool, Taiwan) (Readioff et al., 2020b).

The cross-sectional areas of all ligament specimens were obtained using a previously described method (Goodship & Birch, 2005; Readioff et al., 2020b). In brief, ligaments were encased in a fast-setting alginate impression paste (UnoDent, Essex, England) (Figure 1B). Once the impression material was set, a scalpel blade was used to slice the mould, then filled with polymethyl-methacrylate (PMMA) (Teknovit 6091, Heraeus Kulzer GmbH, Wehrheim, Germany) to create a replica of the ligament structure. Once the PMMA was set, the mould was sliced transversely, and the resulting ends were coloured with a permanent white marker pen (Figure 1C). The cement mould ends were then photographed and digitally measured using ImageJ (Schneider, Rasband & Eliceiri, 2012) to obtain the cross-sectional area of the ligament.

### Specimen preparation

**FIGURE 2:**
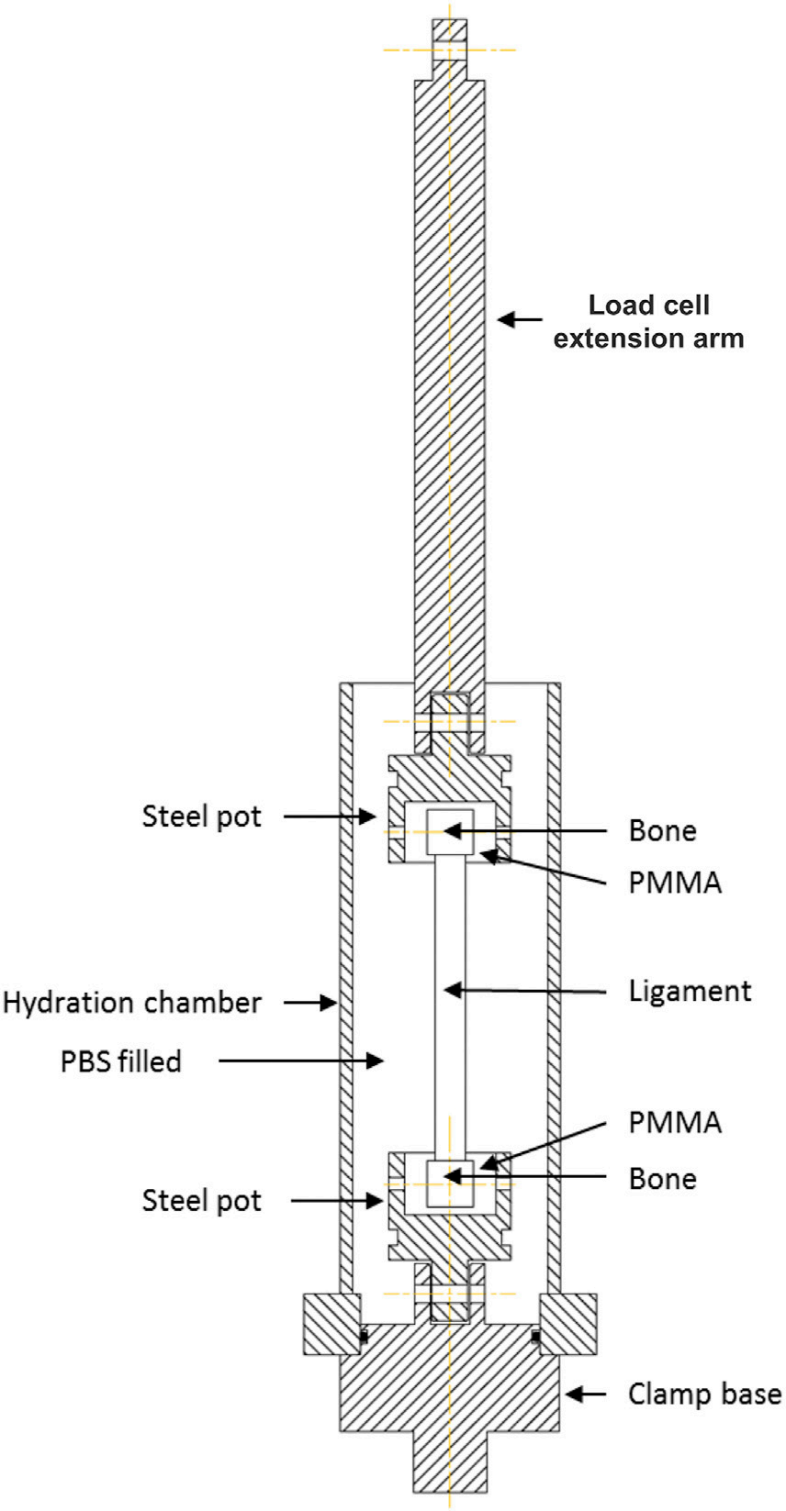
Schematic illustration of the custom-made rig for tensile testing of human knee joint ligaments. The bone ends of the ligament were secured by potting them into a polymethyl-methacrylate (PMMA) holder. Ligaments were encased into a watertight chamber filled with phosphate buffer saline (PBS) to maintain tissue hydration during mechanical tests.

The femur and tibia bones around the attachment sites of each ligament were cut into a suitable shape, maintaining ligaments’ *in vivo* orientation, using a hand saw (Figure 1A). For example, the bone ends of the ACLs and PCLs were cut into a suitable shape to preserve the ligaments’ slight proximal-to-distal spiral during potting. The bone ends of the specimens were potted into custom-made stainless-steel holders and screwed in place. PMMA was then poured into the holder and left to cure for 4–5 min (Comerford et al., 2005; Bonner et al., 2015; Liu et al., 2020). Specimens were then attached to the load cell and encased into a watertight custom-made chamber. The chamber was filled with PBS to control specimen hydration during testing (Figure 2).

### Tensile testing protocol

A uniaxial tensile testing machine (Instron 3366, Buckinghamshire, UK) with a 5000 N load cell (Instron 2519) was used. A preload of 1 N was applied. All ligaments underwent ten preconditioning cycles at 10 mm/min with a load of 1–40 N, which provides a stable and repeatable viscoelastic response (Momersteeg et al., 1995). Loading was then set to zero, and ligaments were loaded to failure at 500 mm/min. A fast strain rate was chosen over slow stain rates to mimic the physiological loading (Noyes & Grood, 1976; Sharma et al., 2008; Bersini, Sansone & Frigo, 2016) and replicate a realistic injury environment (Robinson, Bull & Amis, 2005). In addition, faster strain rates improve the chances of the ligament rupturing mid-substance instead of a bony avulsion (Noyes & Grood, 1976).

### Material properties

**FIGURE 3:**
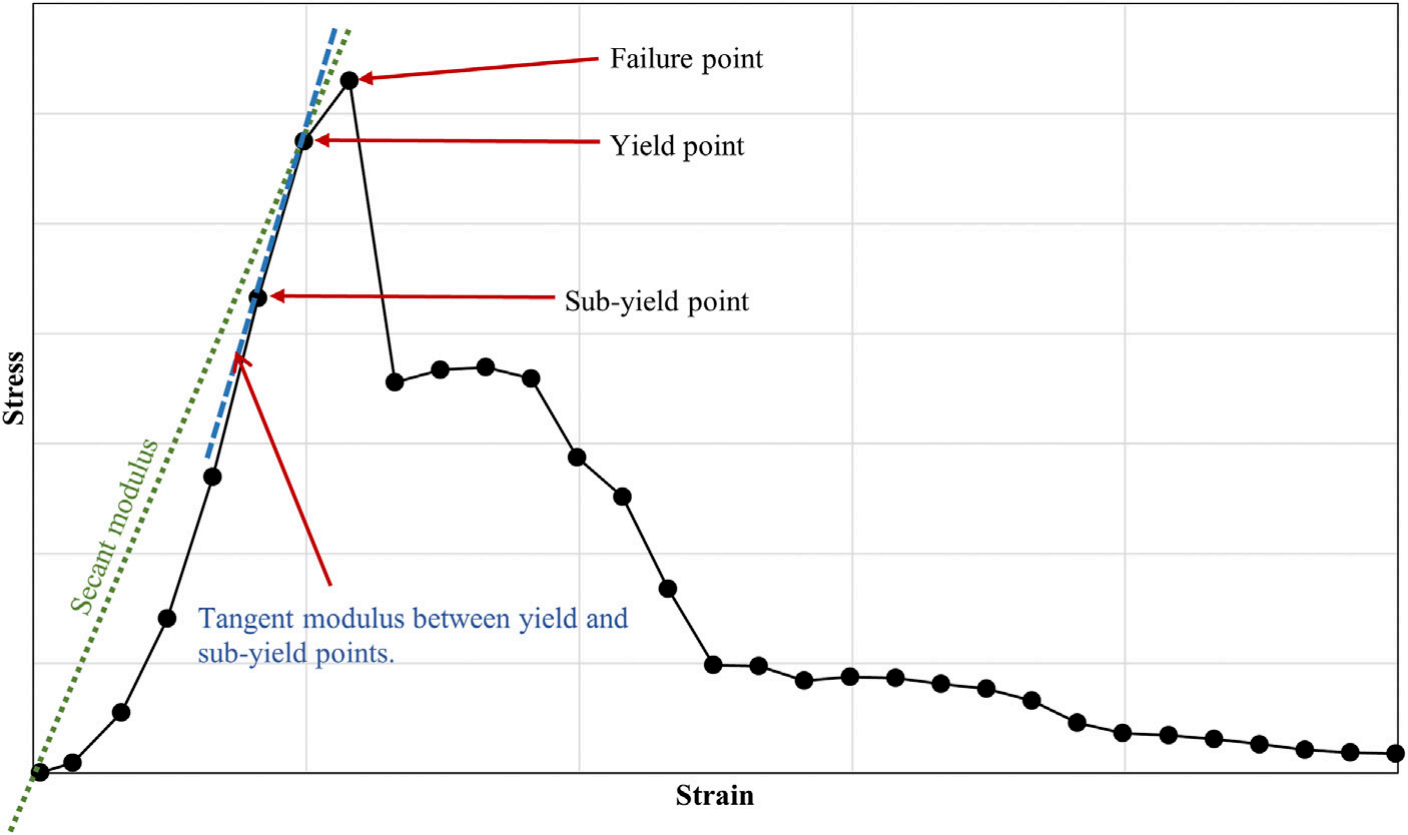
Example of a stress-strain curve, showing failure, yield and sub-yield points in a human knee joint ligament. The figure also highlights the secant and tangent moduli.

The bone-ligament-bone specimens were mechanically tested and analysed to collate multiple material property data. Parameters were obtained from the stress-strain curves, including yield and failure stresses and strains, tangent (the slope between yield and sub-yield) and secant moduli, and stiffness (Figure 3)

$$\sigma_{yield}=\;\frac{F_{yield}}{CSA}$$ (1)

$$\sigma_{sub-yield}=\;\frac{F_{sub-yield}}{CSA}$$ (2)

$$\sigma_{failure\;}=\;\frac{F_{failure}}{CSA}$$ (3)

where $\sigma_{yield}$ , $\sigma_{sub-yield}$ and $\sigma_{failure\;}$ are stresses (MPa) at the yield, sub-yield and failure points, $F_{yield}$ , $F_{sub-yield}$ and $F_{failure}$ are forces (N) at the yield, sub-yield and failure points, and CSA is cross-sectional area (mm^2^).

$$\varepsilon_{yield\;}=\;\frac{L_{yield}-L_{0}}{L_{0}}$$ (4)

$$\varepsilon_{sub-yield\;}=\;\frac{L_{sub-yield}-L_{0}}{L_{0}}$$ (5)

$$\varepsilon_{failure\;}=\;\frac{L_{failure}-L_{0}}{L_{0}}$$ (6)

where $\varepsilon_{yield}$ , $\varepsilon_{sub-yield}$ and $\varepsilon_{failure}$ are strains (%) at the yield, sub-yield and failure points, $L_{yield}$ , $L_{sub-yield}$ and $L_{failure}$ are lengths (mm) at the yield, sub-yield and failure points, and $L_{0}$ is the original length (mm) of the ligament.

$$E_{secant}=\;\frac{\sigma_{yield}}{\varepsilon_{yield\;}}$$ (7)

$$E_{tan}=\;\frac{\sigma_{yield}-\sigma_{sub-yield\;}}{\varepsilon_{yield\;}-\varepsilon_{sub-yield\;}}$$ (8)

$$k\;=\;\frac{E_{secant}\times CSA}{L_{0\;}}$$ (9)

where $E_{secant}$ is secant modulus (MPa), $E_{tan}$ is tangent modulus (MPa) between yield and sub-yield points of the stress-strain curve, and k  is ligament stiffness (N/mm).

### Statistical analysis

Kruskal–Wallis one-way ANOVA was conducted to compare mean differences of ligament material properties between young healthy (≤60 years old, ICRS grade 0), young OA (≤60 years old, ICRS grade 1–4) and old OA (>60, ICRS grade 1–4) cohorts. Ligament tensile properties were correlated with increasing age and grade of OA using Kendall’s Tau-b ( $\tau_{b}$ ) correlation coefficient. The material properties included in the analyses were: yield force, yield stress, yield strain, failure load, failure stress, failure strain, secant modulus, tangent modulus, and stiffness. The statistical analyses were performed in SPSS (SPSS software, Version 22.0, SPSS, Inc., Chicago, IL). For all statistical analyses, the significant level was set at 95% confidence interval (*p* ≤ 0.05).

## Results

### Specimens

ACL (n = 12), PCL (n = 12), MCL (n = 12), and LCL (n = 12) specimens were obtained from twelve human cadavers. One MCL specimen from a young (37 years old) healthy donor was visually determined as severely abnormal, and data from an MCL specimen from ICRS grade 1 donor was unable to be retained (Supplementary Figure S1). Hence, they were excluded from statistical analyses.

The ICRS gradings for all 12 cadaveric knees were given and reported in Table 1, and they are the same as those reported in our previous work (Peters et al., 2018b). Three knees were given ICRS grade 0 (age: 31, 37 and 43 years old), another three knees with ICRS grade 1 (age: 49, 51 and 86 years old), two knees with ICRS grade 2 (age: 58 and 79 years old), three knees with ICRS grade 3 (age: two 72 and 88 years old) and one knee with ICRS grade 4 (age: 80 years old).

**TABLE 1:** Anterior (ACL) and posterior (PCL) cruciate ligaments, medial (MCL) and lateral (LCL) collateral ligaments material property data for all cadavers. ABBREVIATIONS: F, female; M, Male; OA ICRS, Osteoarthritis International Cartilage Repair Society; $L_{0}$ , the original length of the ligament; CSA , cross-sectional area; σ , stress; ε , strain at the yield (_yield_) and failure (_failure_) points of the stress-strain curve; $E_{secant}$ , secant modulus; $E_{tan}$ , tangent modulus; k , stiffness.

Age (years)	Sex	OA ICRS grade	Ligament	$L_{0}$ (mm)	CSA (mm^2^)	$\sigma_{\mathrm{yield}}$ (MPa)	$\varepsilon_{\mathrm{yield}}$ (%)	$E_{\mathrm{secant}}$ (MPa)	$E_{\tan}$ (MPa)	$\sigma_{\mathrm{failure}}$ (MPa)	$\varepsilon_{\mathrm{failure}}$ (%)	k (N/mm)
**31**	F	0	ACL	40	63.80	9.01	12.40	29.06	171.04	9.87	14.48	46.36
			PCL	36	86.53	7.22	12.05	21.57	238.72	10.75	23.62	51.84
			LCL	62	17.10	27.93	17.71	97.79	95.80	31.92	19.05	26.98
			MCL	103	24.51	13.56	6.54	213.56	85.96	17.89	8.16	50.82
37*	F	0	ACL	30	48.46	27.76	22.50	37.03	239.08	32.07	28.05	59.81
			PCL	30	48.82	22.69	32.41	21.00	293.42	27.37	35.19	34.17
			LCL	55	71.75	3.91	14.34	15.02	70.23	6.69	20.40	19.59
			MCL	40	18.19	1.83	10.15	7.19	14.32	2.63	12.24	3.27
**43**	F	0	ACL	32	71.27	6.56	18.08	11.61	126.42	8.09	31.10	25.86
			PCL	30	76.53	4.82	22.78	6.35	73.99	15.95	50.56	16.21
			LCL	61	12.83	6.35	7.19	53.89	34.31	33.25	18.12	11.33
			MCL	108	28.29	12.13	10.34	126.76	75.41	25.72	18.05	33.20
**49**	M	1	ACL	40	34.68	6.61	17.17	15.40	120.79	9.83	23.42	13.35
			PCL	44	84.79	9.17	13.55	29.79	229.97	11.36	21.12	57.41
			LCL	52	39.91	7.00	30.55	11.92	67.32	10.99	41.76	9.15
			MCL	101	22.37	6.15	10.81	57.44	35.88	24.68	17.41	12.72
**51**	M	1	ACL	28	53.64	4.66	17.48	7.46	53.79	9.41	44.27	14.29
			PCL	34	59.24	6.38	14.38	15.10	120.23	17.77	46.24	26.31
			LCL	47	45.15	7.57	14.97	23.77	81.27	9.29	32.70	22.83
			MCL	114	41.18	6.78	6.42	120.38	61.30	8.61	12.27	43.48
**58**	M	2	ACL	41	95.79	1.77	17.00	4.28	55.47	6.93	35.29	10.00
			PCL	46	98.67	9.93	14.99	30.49	220.75	13.99	22.23	65.41
			LCL	58	36.03	10.77	10.14	61.60	103.30	17.47	15.89	38.27
			MCL	127	28.80	5.21	6.18	107.01	30.76	17.85	11.43	24.27
**72 (1)**	M	3	ACL	34	49.75	11.44	11.66	33.39	197.08	16.25	16.56	48.85
			PCL	41	62.51	5.52	18.71	12.09	79.10	8.40	28.88	18.43
			LCL	60	66.07	3.81	16.46	13.88	51.08	5.75	26.18	15.28
			MCL	121	33.41	7.06	6.88	124.16	71.24	13.72	10.32	34.28
**72 (2)**	M	3	ACL	29	101.84	2.61	8.10	9.33	189.12	6.03	16.72	32.76
			PCL	31	91.34	11.60	16.23	22.15	321.15	15.69	21.60	65.28
			LCL	68	44.46	6.35	10.55	40.89	66.36	7.93	16.68	26.73
			MCL	110	58.68	2.05	3.31	68.15	62.58	3.87	12.40	36.35
**79**	M	2	ACL	32	37.78	3.84	23.21	5.30	46.39	4.97	33.63	6.25
			PCL	32	70.34	5.06	17.54	9.24	100.22	9.14	38.37	20.31
			LCL	62	18.98	28.03	14.21	122.29	87.79	33.08	19.59	37.44
			MCL	120	39.57	6.70	14.89	53.98	51.74	8.26	16.27	17.80
**80**	M	4	ACL	38	74.89	1.86	14.91	4.74	77.25	4.99	23.68	9.35
			PCL	35	154.25	0.43	13.98	1.07	29.79	1.70	44.94	4.72
			LCL	74	50.01	5.01	15.74	23.54	59.90	8.91	29.26	15.91
			MCL	116	27.62	14.45	6.62	253.25	73.99	17.84	10.93	60.29
86**	F	1	ACL	30	24.98	3.37	8.58	11.78	58.95	5.38	19.69	9.81
			PCL	43	66.54	1.02	10.89	4.02	40.68	4.22	32.21	6.23
			LCL	60	14.17	6.33	11.52	32.98	33.56	18.58	22.63	7.79
**88**	M	3	ACL	33	64.32	2.25	15.68	4.72	58.76	4.31	28.31	9.21
			PCL	34	95.25	2.23	18.60	4.08	91.30	4.35	28.41	11.43
			LCL	58	24.78	4.12	11.10	21.50	31.79	13.62	24.03	9.19
			MCL	120	35.55	6.55	7.96	98.69	41.48	12.49	14.21	29.24

*Donor had a severely abnormal MCL, and was not included in the statistical analysis.

**The MCL, from this doner could not be retained for mechanical tests.

### Cross-sectional area and length measurements

**FIGURE 4:**
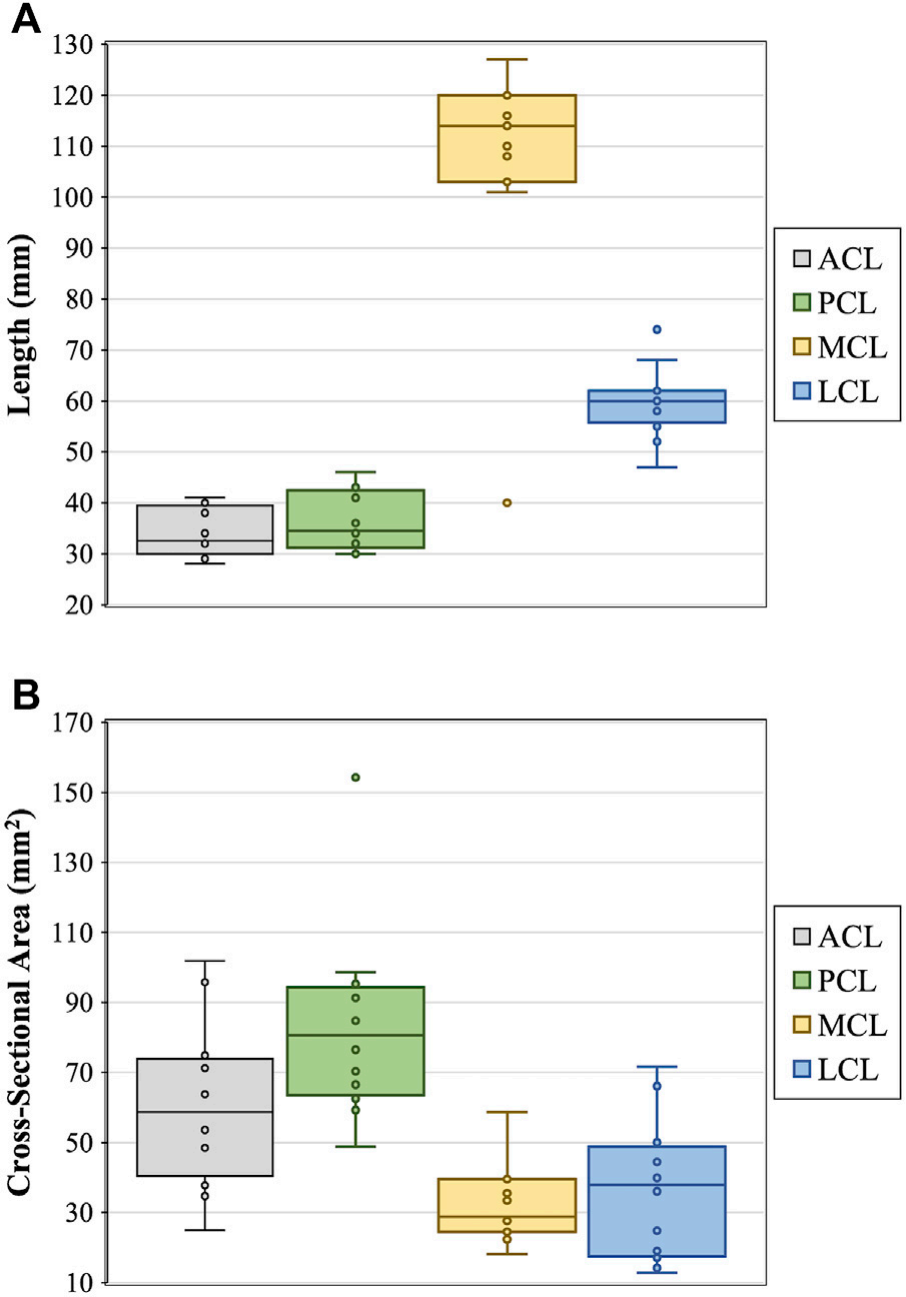
Measurements of ligament **(A)** length and **(B)** cross-sectional area for the anterior (ACL) and posterior (PCL) cruciate ligaments, and medial (MCL) and lateral (LCL) collateral ligaments.

Cross-sectional areas of the ACLs, PCLs, LCLs and MCLs were in the range of 25–102, 49 to 154, 13 to 72, and 18–59 mm^2^, respectively. Lengths of the ACLs, PCLs, LCLs and MCLs were in the range of 28–41, 30 to 46, 47 to 74, and 40–127 mm, respectively. The cross-sectional areas and lengths of individual ligaments for each donor are reported in Table 1 and illustrated in Figure 4.

### Correlation with age

**FIGURE 5:**
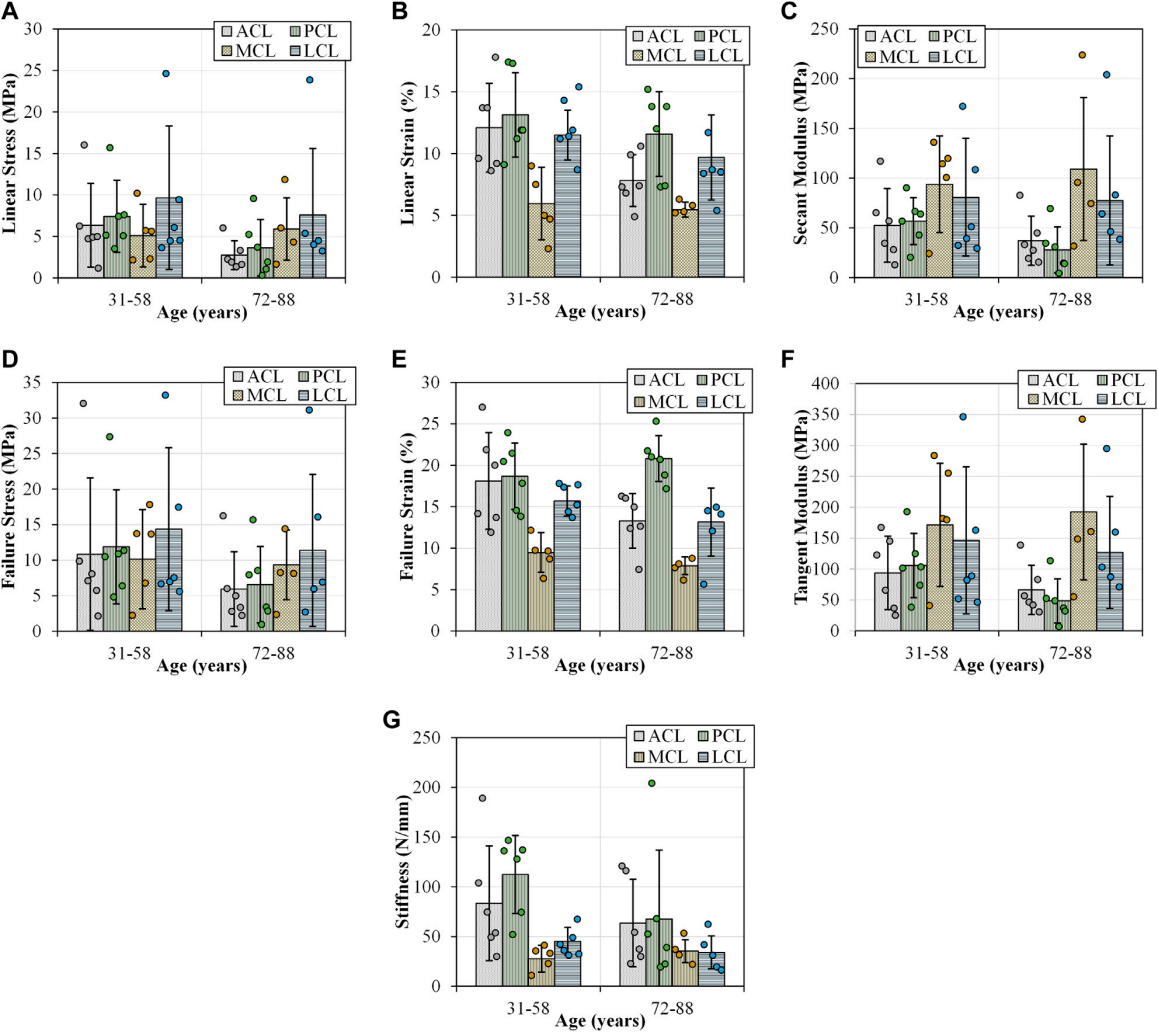
Tensile parameters for the anterior (ACL) and posterior (PCL) cruciate ligaments, and lateral (LCL) and medial (MCL) collateral ligaments across two age groups (31-58 and 72–88 years old). Error bars represent standard deviation. **(A)** Linear stress and **(B)** linear strain were utilised to determine **(C)** secant modulus. **(D)** and **(E)** demonstrates the maximum stresses and strains that resulted in ligament failures. **(F)** This sub-figure shows tangent modulus of the ligaments between the two age groups at the maximum linear region of load-extension curves. **(G)** This sub-figure documents the change in stiffness of the ligaments with age.

Increasing age resulted in statistically significant negative correlations with ACL yield force ( $\tau_{b}$ *= -0.63, p = 0.01*), yield stress ( $\tau_{b}$ *= -0.47, p = 0.03*), yield extension ( $\tau_{b}$ *= -0.44, p = 0.046*), failure force ( $\tau_{b}$ *= -0.50, p = 0.02*) and failure stress ( $\tau_{b}$ *= -0.53, p = 0.02*) (Figure 5). There was no statistically significant correlation between age and ACL yield strain, secant and tangent moduli, failure strain and stiffness (Supplementary Table S4 and ACL_AllStat.xls).

Increasing age showed statistically significant negative correlations with PCL secant modulus ( $\tau_{b}$ *= -0.50, p = 0.02*) and tangent modulus ( $\tau_{b}$ *= -0.53, p = 0.02*). No statistically significant correlations were found between age and the rest of the PCL tensile parameters (Supplementary Table S4 and PCL_AllStat.xls).

There were no statistically significant correlations between age and MCL tensile parameters (MCL_AllStat.xls). Only LCL stiffness showed a statistically significant negative correlation with age ( $\tau_{b}$ *= -0.44, p = 0.046*) and no additional significant correlations were found for the LCL tensile properties (Supplementary Table S4 and LCL_AllStat.xls).

A detailed correlation of age with material properties of the four ligaments is reported in the (Supplementary Table S3, Supplementary Table S4 and Supplementary Figure S3).

### Correlation with osteoarthritis

Increasing OA grade showed a statistically significant negative correlation with ACL yield force ( $\tau_{b}$ *= -0.46, p = 0.048*), yield stress ( $\tau_{b}$ *= -0.53, p = 0.02*), yield extension ( $\tau_{b}$ *= -0.59, p = 0.01*) and yield strain ( $\tau_{b}$ *= -0.5, p = 0.03*). However, the correlations between OA and the rest of the ACL tensile parameters were not statistically significant (Supplementary Table S4 and ACL_AllStat.xls).

No statistically significant correlations existed between OA grade and PCL and MCL tensile parameters. Only LCL failure stress showed a statistically significant negative correlation between OA grade and LCL failure stress ( $\tau_{b}$ *= -0.46, p = 0.048*), and the rest of the LCL tensile parameters were not statistically significant (PCL_AllStat.xls, MCL_AllStat.xls, and LCL_AllStat.xls).

A detailed correlation of OA with material properties of the four ligaments is reported in the (Supplementary Table S3, Supplementary Table S4 and Supplementary Figure S3).

## Discussion

This paper reports the first *ex vivo* study to quantify the effects of ageing and OA on the material properties of the four primary knee ligaments from the same cadaveric joints within a wide span of age (31–88 years old) and OA grade (ICRS 0–4). Our results showed statistically significant negative correlations with ACL yield and failure forces, stresses and extensions, PCL secant and tangent modulus and LCL stiffness (Supplementary Table S4). Similarly, increasing OA grade showed a statistically significant negative correlation with ACL yield forces, stresses, extensions, strains, and LCL failure stress (Supplementary Table S4). Changes in age or OA grade did not significantly correlate with the MCL material parameters (Supplementary Table S4). This data is vital for understanding joint mechanics, and it can provide an insight into the progression of OA as a whole-joint disease as well as the effects of ageing, notably because bone and cartilage mechanical properties for these specific human cadavers have already been reported in our previous study (Peters et al., 2018b).

Failure loads previously reported across any age category span two orders of magnitude between 495 and 2160 N in the ACL, 258–1620 N in the PCL, 194–534 N in the MCL and 376 N in the LCL (Noyes & Grood, 1976; Trent, Walker & Wolf, 1976; Woo et al., 1991; Race & Amis, 1994; Harner et al., 1995; Chandrashekar et al., 2006). Furthermore, previous studies also reported stiffness values which ranged between 124 and 308 N/mm in the ACL, 57–347 N/mm in the PCL, 70 N/mm in the MCL and 59 N/mm in the LCL, where values reported for failure load (Figure 5D) and stiffness (Figure 5G) in the current study fall within the previously reported range (Noyes & Grood, 1976; Trent, Walker & Wolf, 1976; Woo et al., 1991; Race & Amis, 1994; Harner et al., 1995; Chandrashekar et al., 2006). Previous research has indicated a decrease in the ACL failure load with increasing age, consistent with the current study (Figure 5D). Age-based differences show ACL failure loads of up to 2160 N amongst younger donors (22–35 years), 1503 N in middle-aged donors (40–50 years) and 658 N amongst older donors (60–97 years). However, Woo et al. (1991) did not indicate degeneration of joint integrity.

**FIGURE 6:**
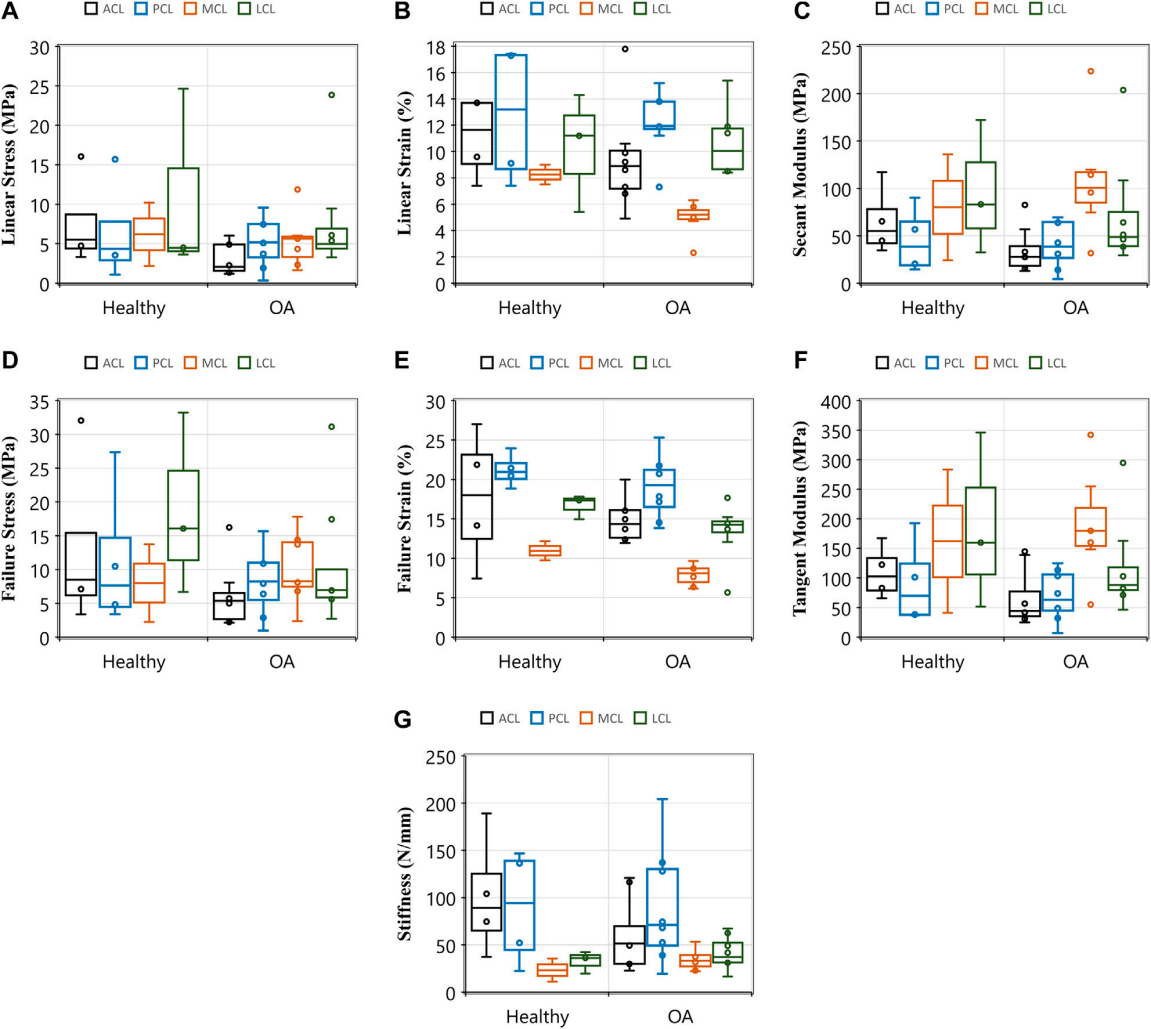
Comparisons of tensile properties of the anterior (ACL) and posterior (PCL) cruciate ligaments, and lateral (LCL) and medial (MCL) collateral ligaments between healthy and osteoarthritic (OA) groups. Healthy groups were defined by International Cartilage Repair Society (ICRS) grade 0 and osteoarthritic (OA) was defined by ICRS grade 1-4. **(A)** Linear stress and **(B)** linear strain were utilised to determine **(C)** secant modulus. **(D)** and **(E)** demonstrates the maximum stresses and strains that resulted in ligament failures. **(F)** This sub-figure shows tangent modulus of the ligaments between the healthy and OA groups at the maximum linear region of load-extension curves. **(G)** This sub-figure documents ligament stiffness values across ligaments and between the healthy and OA groups.

The current research showed a decrease in the failure strain of all four knee ligaments with the development of OA (Figure 6E and Supplementary Figure S3). The ACL in healthy knees showed higher yield and failure stresses (Figures 6A,D) and strains (Figures 6B,E), secant (Figure 6C) and tangent (Figure 6F) modulus and stiffness (Figure 6G) when compared to those with OA. The influence of OA has previously been investigated in animal models, and a reduction in tensile properties of the rat ACL was reported 10 weeks after collagen-induced arthritis. Ultimate failure load was reduced by 25.1% and stiffness by 38.0% compared to controls (Nawata et al., 2001). Despite a lack of knee joint material properties in the literature associated with OA in humans, previous research has found that between 39 and 78% of patients with OA have a degenerated ACL (Allain, Goutallier & Voisin, 2001; Cushner et al., 2003; Lee et al., 2005; Mullaji et al., 2008; Watanabe et al., 2011), and between 7 and 80% have a degenerated PCL (Nelissen & Hogendoorn, 2001; Stubbs et al., 2005; Mullaji et al., 2008). Such degeneration is consistent with the decrease in our current study’s tensile properties of the four knee joint ligaments.

It is challenging to separate the effects of OA and ageing as they often happen concurrently. With only 12 cadavers and five groups of ICRS grades (0–4), it was challenging to statistically attribute changes in ligament tensile properties to both age and OA as related parameters, mainly when also accounting for sex (see further discussion below). However, trends were analysed from the data presented in Table 1 to understand the effect of age and OA as individual parameters. The trends suggest that the ACL and PCL material properties in younger donors were reduced in the OA knees compared to those in the healthy knees (Supplementary Figure S4). The findings imply that even mild OA in younger donors affects the material properties further exacerbated with advancing age and OA.

OA is believed to be a whole-joint disease impairing the integrity of associated tissues, including ligaments (Poole, 2012). In our previous study on the same human cadaveric knees, we found statistically significant correlations between changes in material properties of cartilage and subchondral bone with age and OA grade (Peters et al., 2018b). Similarly, the data in the current study for the same cadavers showed alterations in ligament tensile properties because of OA. Ligament degeneration or injury may occur in the first instance, leading to the initiation and progression of knee OA (Gianotti et al., 2009). Since the primary function of knee ligaments is to provide stability to the knee joint (Harner et al., 1995; Woo et al., 2006), any changes to the ligaments’ structure can alter the load distribution in the knee joint (Moore & Burris, 2015). The knee cadavers in this study showed that OA degeneration affected the medial more than the lateral compartments of the bones (Peters et al., 2018b). The difference in degeneration between the lateral and medial compartments of the knee joint could result from unbalanced load distribution caused by changes in the ligament material properties because of OA.

The reduction in the measured tensile parameters of the ACL during ageing and disease progression may be attributed to the relatively high forces experienced during walking. There is a consensus that peak force experienced by the ACL occurs at the contralateral toe-off during the stance phase of the gait cycle, up to 3.5 times body weight (Morrison, 1970; Collins & O’Connor, 1991; Shelburne et al., 2004). In particular, these high ACL kinematic forces may be consistent with the widely reported histological degeneration of the ACL in the presence of disease (Mullaji et al., 2008), suggesting high habitual forces could influence subsequent degeneration observed. The peak force of the PCL has also been reported to be 0.2 to 0.6 times body weight during walking (Morrison, 1970; Collins & O’Connor, 1991). Evidence shows that appropriate exercise training strengthens ligaments and knee joint mechanics (Tipton et al., 1975; Salem et al., 2000; Ng, 2002; Ferri et al., 2003). However, people exercise less as they age, increasing their risk of ligament degeneration (Daley & Spinks, 2000). Decreased capacity of the knee ligaments to resist motion due to reduced mechanical strength may alter joint contact forces, potentially causing increased loading on the medial femoral condyle and contributing to the preferential medial development of OA (Lohmander et al., 2007; Pelletier et al., 2007).

Further limitations of the current study, aside from a low sample number, include varying donor demographics, such as sex, which is known to affect tensile properties and the likelihood of knee ligament injury. It was found that ACLs in young human females had 22.49% lower Young’s modulus, and 8.3% and 14.3% lower failure strain and stress, respectively, compared to ACLs in young human males (Chandrashekar et al., 2006). These differences can be partially attributed to the physically smaller size of the female ACL, which can be linked to higher rates of ACL injuries in female athletes (Anderson et al., 2001; Chandrashekar, Slauterbeck & Hashemi, 2005). Human females are also at a greater risk of knee OA than their male counterparts (Hame & Alexander, 2013). Again, this study could not separate ligaments by sex for statistical analyses due to low sample numbers.

Finally, the current study may be limited by testing ligaments as whole bone-ligament-bone specimens along their loading axis. It has previously been acknowledged that ligaments may be best divided into fibre bundles to recruit fibres to their maximal potential and eliminate any slack due to orientation (Woo et al., 1991; Race & Amis, 1994). Significant differences have been reported between the anterior and posterior fibres of the ACL (Butler et al., 1992) and PCL (Race & Amis, 1994; Harner et al., 1995), suggesting that fibres play different roles in the stabilisation of the knee joint (Race & Amis, 1994); although ligaments naturally work as one functional unit. Such global approaches have been used to represent ligaments in finite element models as one functional unit (Readioff et al., 2020a). However, due to the lack of data on all four ligaments from the same donor (and in some instances, the same demographic or disease conditions of the donor) in the literature, material properties have often been applied globally in finite element models, where values for one ligament are replicated for all others (Blankevoort & Huiskes, 1991; Li, Lopez & Rubash, 2001; Kazemi & Li, 2014; Wang, Fan & Zhang, 2014). In some instances, tendon material properties have been used (Kazemi et al., 2011; Kazemi & Li, 2014; Wang, Fan & Zhang, 2014). Sensitivity analysis showed that varying intrinsic ligament material properties alter the internal and external rotation of the tibia-femoral joint, patella tilt and peak contact stress (Dhaher, Kwon & Barry, 2010). The data in this study, combined with cartilage and bone data in our previous study (Peters et al., 2018b), allows future research to apply a subject- or cohort-specific approach to computational modelling of the human knee joint to improve accuracy and predictive behaviour patterns of ligaments.

The knowledge of baseline material properties of all four ligaments from healthy donors can be used to replicate ligaments by developing more biologically representative synthetic materials for the repair and replacement following injury or degeneration (Yang, Rothrauff & Tuan, 2013; Ratcliffe et al., 2015). Future studies could investigate the effect of ageing and osteoarthritis on viscoelastic characteristics (creep and stress-relaxation) and biochemical composition (Kharaz et al., 2018) of these ligaments in the same knee. The data collected in this study provides insight into the healthy range for these parameters and how they change concurrently with surrounding ligaments during ageing and disease.

## Conclusion

This research is the first to report material characteristics of the four major human knee ligaments from a diverse demographic such as healthy, aged, and osteoarthritic knees. We confirmed previous research findings that the ACL tensile properties decrease with age and OA. The results also showed that the PCL tangent and secant modulus decrease with increasing age. These data and our previously reported data on bone and cartilage material properties for the same cadavers support current research stating that OA is a whole-joint disease impairing many peri-articular tissues within the knee. The material properties of the four major knee ligaments in the twelve cadavers can be combined with their corresponding subchondral and trabecular bones and articular cartilage for future subject-specific applications, including the development of computational models.

## Data Availability

The original contributions presented in the study are included in the article/Supplementary Material, further inquiries can be directed to the corresponding authors.
